# Impact of coronary revascularization on clinical outcomes of postacute myocardial infarction patients with left ventricular thrombus

**DOI:** 10.1016/j.rpth.2025.102897

**Published:** 2025-05-21

**Authors:** Andre Wen–Jie Seah, Aloysius Sheng–Ting Leow, Fang–Qin Goh, Benjamin Yong–Qiang Tan, Leonard Leong–Litt Yeo, William K.F. Kong, Kian–Keong Poh, James W.L. Yip, Raymond Ching–Chiew Wong, Ping Chai, Tiong–Cheng Yeo, Mark Yan–Yee Chan, Xin Zhou, Gregory Y.H. Lip, Ching–Hui Sia

**Affiliations:** 1 Department of Medicine, National University Health System, Singapore; 2 Department of Medicine, Yong Loo Lin School of Medicine, National University of Singapore, Singapore; 3 Division of Neurology, Department of Medicine, National University Health System, Singapore; 4 Department of Cardiology, National University Heart Centre Singapore, Singapore; 5 Department of Cardiology, Tianjin Medical University General Hospital, Tianjin, China; 6 Liverpool Centre for Cardiovascular Science, University of Liverpool, Liverpool John Moores University, Liverpool Heart and Chest Hospital, Liverpool, UK; 7 Department of Clinical Medicine, Danish Center for Health Services Research, Aalborg University, Aalborg, Denmark

**Keywords:** acute coronary syndrome, anticoagulation, left ventricular thrombus, revascularization, stroke

## Abstract

**Background:**

The incidence of left ventricular thrombus (LVT), a significant complication postacute myocardial infarction (AMI), has seen a decline in the percutaneous coronary intervention era. Patients may not undergo coronary revascularization due to medical contraindications or patient preference.

**Objectives:**

This study compared post-AMI LVT patients treated with or without revascularization.

**Methods:**

This was a retrospective study of 263 consecutive post-AMI patients diagnosed with LVT from November 2012 to January 2021, retrieved from an echocardiography database. Patients were stratified by their revascularization status.

**Results:**

Mean (SD) follow-up duration was 2.1 ± 2.1 years. Most post-AMI LVT patients underwent revascularization via percutaneous coronary intervention (71.5%; *n* = 188). Unrevascularized patients (24.0%; *n* = 63) were older (*P* < .001), more often female (*P* < .001), more comorbid, less likely to have anterior AMI (*P* < .001), or treated with anticoagulation (*P* < .001). In multivariable analysis, at least anticoagulation + P2Y12 inhibitor (adjusted hazard ratio [aHR], 1.84; 95% CI, 1.14-2.96; *P* = .01), but not revascularization (aHR, 1.25; 95% CI, 0.74-2.13; *P* = .40), was associated with LVT resolution. Both absence of revascularization (aHR, 2.30; 95% CI, 1.09-4.85; *P* = .03) and LVT resolution (aHR, 6.06; 95% CI, 2.99-12.3; *P* < .001) were associated with higher mortality after adjusting for age, sex, anemia, anterior AMI, and ejection fraction.

**Conclusion:**

Lack of revascularization in post-AMI LVT patients was associated with higher mortality but not LVT resolution. Optimizing medical therapy remains a key treatment goal.

## Essentials

• Left ventricular thrombus (LVT) formation is a significant complication in postacute myocardial infarction (AMI) patients. The impact of revascularization in such patients is unclear.
• This was a retrospective study from a single center of 263 consecutive post-AMI patients with LVT from 2012 to 2021 in an echocardiography database, stratified by revascularization status.
• Lack of revascularization in post-AMI LVT patients was associated with higher mortality but not LVT resolution.
• Optimizing medical therapy remains a key treatment goal.

## 1 Introduction

Left ventricular thrombus (LVT) is a common complication in postacute myocardial infarction (AMI) patients and can be a significant source of morbidity and mortality, primarily due to arterial thromboembolism such as acute ischemic stroke and acute limb ischemia [[1], [2], [3], [4]]. The post-AMI state provides a favorable milieu for the formation of LVT by the combination of stunned myocardium or regional wall motion abnormalities, a proinflammatory state triggered by tissue necrosis, and endothelial damage directly from myocardial infarction (MI), which corresponds with Virchow’s triad of thrombosis.

Reported incidence of LVT in post-AMI patients was between 1.6% and 46% [[5], [6], [7], [8]] in the prereperfusion era compared with the much lower 3% to 15% [1] in the primary percutaneous coronary intervention (pPCI) era. The emphasis on rapid reperfusion and timely revascularization [[9], [10], [11]] in the pPCI era resulted in a shorter ischemia time, improving myocardial salvage and minimizing the size of the infarcted area. Thus, the decrease in incidence of LVT may be attributed to smaller infarctions, improved left ventricular (LV) remodeling post-AMI, and earlier anticoagulation [12].

Current European and American guidelines recommend that a period of anticoagulation, along with dual antiplatelet therapy (DAPT), termed triple therapy, should be initiated in post-AMI patients with concomitant LVT for a duration of 1 to 4 weeks prior to switching to dual therapy [13,14]. However, the contribution of revascularization itself to the decreased incidence of LVT is unclear, and other factors such as earlier diagnosis with guideline-recommended screening echocardiography, improved echocardiography, and earlier anticoagulation or improvements in medical therapy post-MI may account for this change. While multiple studies have reported on the effects of anticoagulation on clinical outcomes in LVT patients [15], there remains a paucity of data in the current literature examining the effects of revascularization on post-AMI LVT patients. This is an important area of study that may have been overlooked in the contemporary pPCI era, as a small but significant minority of patients with AMI still do not undergo revascularization either due to medical contraindications or patient preference.

Hence, the aim of this study was to describe the clinical characteristics of post-AMI patients with LVT who do not undergo revascularization and to evaluate the prognostic effect of revascularization status on clinical outcomes.

## 2 Methods

### 2.1 Study design and population

**Figure 1:**
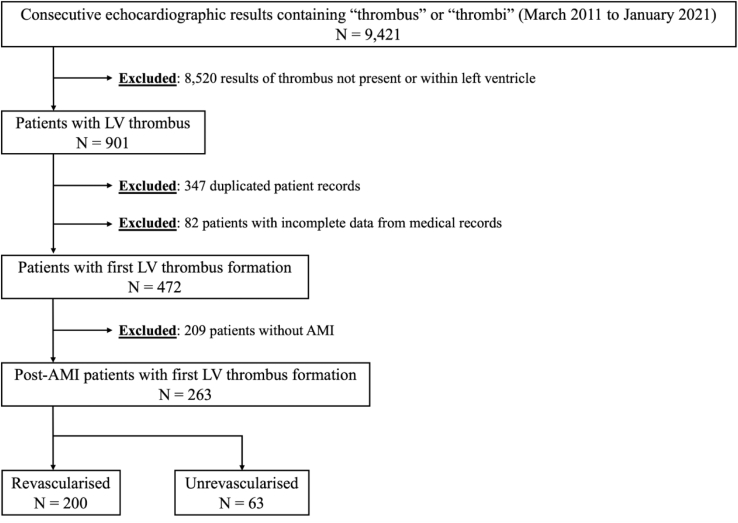
Patient selection flowchart. AMI, acute myocardial infarction; LV, left ventricular.

This was a retrospective observational study of post-AMI patients diagnosed with LVT based on an echocardiography database established from March 2011 to January 2021 within a single tertiary academic medical center. Ethical approval was obtained from the local institutional review board (National Healthcare Group domain-specific review board study number 2021/00066). Figure 1 illustrates the selection of patients for this study. An initial keyword search for “thrombus” or “thrombi” retrieved 9421 consecutive echocardiography reports from the institution’s electronic health records. Subsequently, a rigorous screening process was applied to exclude reports of thrombus not found in the left ventricle (*n* = 8520), duplicated records (*n* = 347), those with incomplete data (such as missing baseline characteristics, insufficient data to diagnose AMI, or unclear revascularization status; *n* = 82), or those without AMI (*n* = 209). Finally, 263 eligible post-AMI patients with confirmed LVT were included and stratified into 2 groups based on the presence or absence of revascularization treatment. Relevant data, such as baseline characteristics, treatment modalities, and outcomes, were collected.

### 2.2 AMI and revascularization

AMI was defined based on the presence of typical symptoms of ischemia, electrocardiographic changes (new ST-T changes and new left bundle branch block), and biomarker evidence (cardiac troponin >99th percentile upper limit) in accordance with the fourth universal definition of MI [14,16].

All AMI patients underwent coronary angiography and subsequent revascularization unless deemed to be medically unsuitable or due to patient preference. Upon diagnosis of AMI, DAPT was initiated with aspirin (loading dose of 300 mg followed by 100 mg/d) and clopidogrel (loading dose of 300 mg or 600 mg followed by 75 mg/d) or ticagrelor (loading dose of 180 mg followed by 90 mg twice a day) in accordance with European Society of Cardiology guidelines [14]. Patients who underwent pPCI received periprocedural intravenous anticoagulation as determined by the interventionist.

### 2.3 Diagnosis and treatment of LVT

All post-AMI patients underwent transthoracic echocardiograms (TTE) conducted by a certified echosonographer during the index admission. The diagnosis of LVT was based on specific criteria: the presence of an echo-dense mass visible in at least 2 different views, adjacent to a myocardial segment with impaired wall motion (hypokinetic or akinetic), well-defined margins distinct from the endocardium, and distinguishable from other intracardiac structures or artifacts [17]. Key information on the LVT, such as size, mobility, protrusion, and presence of LV aneurysm, was reported. LV ejection fraction (LVEF) was calculated using the Simpson Biplane method of disks [18]. All TTE images during the time of reporting were independently evaluated by experienced certified cardiologists. In cases where the initial TTE was equivocal for the presence of LVT, additional scans, including contrast echocardiography or cardiac magnetic resonance (MR) imaging, were performed to confirm its presence. Patients with confirmed LVT were initiated on anticoagulation, with heparin bridging by enoxaparin or continuous heparin infusion followed by oral anticoagulation, targeting a therapeutic international normalized ratio of 2 to 3. Triple therapy was defined as the combination of anticoagulation with DAPT (aspirin and clopidogrel), and dual therapy as combination of anticoagulation with clopidogrel. Follow-up imaging was also conducted to assess the resolution of LVT at 3 to 6 months post-AMI based on institutional protocol.

### 2.4 Study endpoints and statistical analysis

The primary endpoint studied was LVT resolution, while the secondary endpoints included bleeding events, acute ischemic stroke or transient ischemic attack (TIA), and all-cause mortality. Thrombus resolution was defined as the absence of any detectable thrombus during subsequent cardiac imaging. Acute ischemic stroke refers to the emergence of a new-onset neurological deficit, persisting for >24 hours or until death, stemming from ischemic causes and confirmed via neuroimaging methods such as computed tomography or MR imaging [19]. Categorical variables were presented as frequencies and percentages, while continuous variables were presented as means ± SDs. Statistical analyses for categorical variables were performed using chi-squared test and independent sample *t*-test for continuous variables. The Kaplan–Meier method was utilized for survival analysis (specifically for all-cause mortality), and the difference was evaluated via the log-rank test. We accounted for the competing risk of all-cause mortality by utilizing the cumulative incidence function estimates for thrombus resolution, bleeding events, and subsequent stroke/TIA outcomes [20]. Similarly, time-to-event analysis for all-cause mortality was performed using the Cox proportional hazards model, adjusting for age, female sex, anemia, anterior ST-segment elevation MI (STEMI), LVEF, revascularization status, and absence of LVT resolution, which were decided *a priori* based on existing literature, and were presented as hazard ratio (HR) and 95% CI. Multivariate analyses for thrombus resolution, bleeding, and subsequent stroke/TIA were performed with the Fine and Gray competing risks model, considering the frequency of mortality within the post-AMI LVT cohort [21], and were adjusted for age, female sex, anemia, anterior STEMI, LVEF, revascularization status, and at least dual therapy. All *P* values < .05 were considered statistically significant. The statistical analysis was conducted using R statistical software (v4.3.1; R Foundation for Statistical Computing) and RStudio (v2023.12.1; Posit PBC) with the following key packages: ggsurvfit [22] and tidycmprsk [23].

## 3 Results

In this cohort of 263 post-AMI patients with LVT, the mean (SD) age was 58.8 ± 12.9 years, with a smaller proportion of female patients (11.4%; *n* = 30). Most patients underwent revascularization via percutaneous coronary intervention (PCI; 71.5%; *n* = 188), coronary artery bypass graft (4.2%; *n* = 11), or thrombolysis (0.4%; *n* = 1), while a small proportion of patients remained unrevascularized (24.0%; *n* = 63). A comparative analysis of these 2 groups of patients, based on their revascularization status, is presented in Table 1.

**Table 1:** Clinical characteristics of postacute myocardial infarction left ventricular thrombus patients stratified by revascularization status.

Variables	*N*	Overall *N* = 263	Revascularized *n* = 200	Unrevascularized *n* = 63	*P* value
**Patient characteristics**					
Age (y), mean (SD)	263	58.8 (12.9)	56.3 (11.6)	66.7 (13.6)	**<.001**
Female sex, *n* (%)		30 (11.4)	15 (7.5)	15 (23.8)	**<.001**
Race, *n* (%)					.059
Chinese		137 (52.1)	106 (53.0)	31 (49.2)	
Malay		67 (25.5)	44 (22.0)	23 (36.5)	
Indian		42 (16.0)	37 (18.5)	5 (7.9)	
Others		17 (6.5)	13 (6.5)	4 (6.3)	
BMI (kg/m^2^), mean (SD)		25.1 (4.6)	25.6 (4.4)	23.2 (5.0)	**.002**
Comorbidities, *n* (%)					
Current or previous smoker		140 (53.2)	112 (56.0)	28 (44.4)	.109
Atrial fibrillation		5 (1.9)	3 (1.5)	2 (3.2)	.596
Hypertension		123 (46.8)	84 (42.0)	39 (61.9)	**.006**
Hyperlipidemia		121 (46.0)	86 (43.0)	35 (55.6)	.08
Diabetes mellitus		92 (35.0)	63 (31.5)	29 (46.0)	**.035**
Previous stroke or TIA		32 (12.2)	14 (7.0)	18 (28.6)	**<.001**
Known ischemic heart disease		68 (25.9)	50 (25.0)	18 (28.6)	.57
Previous heart failure		26 (9.9)	13 (6.5)	13 (20.6)	**.001**
Previous VTE		2 (0.8)	1 (0.5)	1 (1.6)	.42
Peripheral vascular disease		9 (3.4)	5 (2.5)	4 (6.3)	.22
Chronic kidney disease		29 (11.0)	17 (8.5)	12 (19.0)	**.02**
ESKD		3 (1.1)	1 (0.5)	2 (3.2)	.14
Malignancy		8 (3.0)	3 (1.5)	5 (7.9)	**.02**
Laboratory results, mean (SD)					
Hb (g/dL; N: 13.1-16.6)		14.1 (2.3)	14.4 (2.2)	13.2 (2.4)	**<.001**
TW (×10^9^/L; N: 3.8-10.0)		12.6 (4.5)	12.9 (4.3)	11.8 (4.8)	.107
Plt (×10^9^/L; N: 164-387)		251 (85)	250 (82)	254 (94)	.739
INR		1.2 (0.7)	1.2 (0.8)	1.2 (0.4)	.98
eGFR (mL/min; N: >60)		77.5 (25.8)	81.3 (23.3)	65.2 (29.8)	**<.001**
Cr (μmol/L; N: 60-110)		105 (86)	96 (45)	135 (153)	**.001**
**AMI characteristics**					
Duration from MI (d), mean (SD)	263	3.9 (3.3)	3.9 (3.3)	3.8 (3.3)	.79
NSTEMI, *n* (%)		57 (21.7)	20 (10.0)	37 (58.7)	**<.001**
Anterior STEMI, *n* (%)		206 (78.3)	180 (90.0)	26 (41.3)	**<.001**
Type of revascularization, *n* (%)					**<.001**
None		63 (24.0)	0 (0.0)	63 (100.0)	
PCI		188 (71.5)	188 (94.0)	0 (0.0)	
CABG		11 (4.2)	11 (5.5)	0 (0.0)	
Thrombolysis		1 (0.4)	1 (0.5)	0 (0.0)	
LVEF (%), mean (SD)		34.2 (10.5)	35.6 (10.5)	29.5 (9.0)	**<.001**
LVT features, *n* (%)					
LV aneurysm		31 (11.8)	23 (11.5)	8 (12.7)	.797
Mobility		11 (4.2)	7 (3.5)	4 (6.3)	.30
Protrusion		16 (6.1)	12 (6.0)	4 (6.3)	>.999
Size (cm), mean (SD)		1.5 (0.6)	1.5 (0.6)	1.6 (0.6)	.147
**Treatment**					
GDMT, *n* (%)	253				
ACEi or ARB		156 (61.7)	130 (66.7)	26 (44.8)	**.003**
Beta blockers		210 (83.0)	168 (86.2)	42 (72.4)	**.01**
MRA		42 (16.6)	32 (16.4)	10 (17.2)	.88
Aspirin, *n* (%)	263	214 (81.4)	172 (86.0)	42 (66.7)	**<.001**
Second antiplatelet, *n* (%)		193 (73.4)	170 (85.0)	23 (36.5)	**<.001**
Heparin bridging, *n* (%)					**.03**
LMWH		202 (76.8)	159 (79.5)	43 (68.3)	
UFH		30 (11.4)	23 (11.5)	7 (11.1)	
Antithrombotic strategy, *n* (%)					**<.001**
None		4 (1.5)	1 (0.5)	3 (4.8)	
SAPT only		5 (1.9)	0 (0.0)	5 (7.9)	
DAPT only		6 (2.3)	3 (1.5)	3 (4.8)	
ACC only		25 (9.5)	13 (6.5)	12 (19.0)	
ACC + SAPT		55 (20.9)	30 (15.0)	25 (39.7)	
ACC + DAPT		168 (63.9)	153 (76.5)	15 (23.8)	
Any ACC, *n* (%)		248 (94.3)	196 (98.0)	52 (82.5)	**<.001**
Duration of ACC (y), mean (SD)	248	1.1 (1.4)	1.1 (1.4)	1.2 (1.6)	.607
Duration of triple therapy (y), mean (SD)	162	1.1 (1.5)	1.1 (1.4)	1.6 (2.0)	<.001
**Outcomes**					
Duration of follow-up (y), mean (SD)	263	2.1 (2.1)	2.3 (2.1)	1.5 (1.9)	**.003**
Lost to follow-up, *n* (%)		25 (9.5)	19 (9.5)	6 (9.5)	.996
Final LVEF (%), mean (SD)	178	40.1 (12.3)	40.6 (12.0)	38.1 (13.7)	.359
Change in LVEF (%)		4.7 (10.0)	4.2 (9.8)	7.4 (10.6)	.13
LVT resolution, *n* (%)	222	154 (69.4)	130 (75.6)	24 (48.0)	**<.001**
Recurrence	154	5 (3.2)	3 (2.3)	2 (8.3)	.17
Duration to LVT resolution (y), mean (SD)	154	0.6 (0.7)	0.6 (0.7)	0.7 (0.8)	.76
Major adverse events, *n* (%)	263				
Bleeding outcome		31 (11.8)	23 (11.5)	8 (12.7)	.795
Major bleeding (BARC ≥2)		25 (9.5)	18 (9.0)	7 (11.1)	.618
Stroke outcome		20 (7.6)	15 (7.5)	5 (7.9)	>.999
All-cause mortality		59 (22.4)	28 (14.0)	31 (49.2)	**<.001**
In-hospital mortality		29 (11.0)	15 (7.5)	14 (22.2)	**.001**
Duration to all-cause mortality (y), mean (SD)	263	0.6 (0.9)	0.6 (1.0)	0.5 (0.8)	.59

ACC, anticoagulation; ACEi, angiotensin-converting enzyme inhibitor; AMI, acute myocardial infarction; ARB, angiotensin receptor blocker; BARC, Bleeding Academic Research Consortium; BMI, body mass index; CABG, coronary artery bypass graft; Cr, creatinine; DAPT, dual antiplatelet therapy; eGFR, estimated glomerular filtration rate; ESKD, end-stage kidney disease; GDMT, guideline directed medical therapy; Hb, hemoglobin; INR, international normalized ratio; LMWH, low-molecular-weight heparin; LV, left ventricular; LVEF, left ventricular ejection fraction; LVT, left ventricular thrombus; MI, myocardial infarction; MRA, mineralocorticoid receptor antagonists; N, number; NSTEMI, non–ST-segment elevation myocardial infarction; PCI, percutaneous coronary intervention; Plt, platelet; SAPT, single antiplatelet therapy; TIA, transient ischemic attack; TW, total white blood cell; UFH, unfractionated heparin; VTE, venous thromboembolism.

In contrast to those that underwent revascularization, unrevascularized patients were significantly older (*P* < .001), more likely to be female (*P* < .001), and had a greater burden of comorbidities such as hypertension (*P* = .006), diabetes mellitus (*P* = .035), previous stroke or TIA (*P* < .001), previous heart failure (*P* = .001), chronic kidney disease (*P* = .02), and malignancy (*P* = .02). More unrevascularized patients had non–ST-elevation MI (*P* < .001), and fewer had anterior STEMI (*P* < .001) than revascularized patients. LVEF was significantly lower in unrevascularized patients (29.5% ± 9.0%) than in revascularized patients (35.6% ± 10.5%; *P* < .001), expressed as mean ± standard deviation.

In terms of medical therapy, significantly fewer unrevascularized patients were treated with any anticoagulation (*P* < .001) compared with revascularized patients. Over a mean (SD) follow-up duration of 2.1 ± 2.1 years, unrevascularized patients were observed to have a significantly lower incidence of LVT resolution (*P* < .001) but significantly higher rates of all-cause mortality (*P* < .001) and in-hospital mortality (*P* = .001) compared with revascularized patients. Among revascularized patients, incidence of LVT resolution was similar between those who underwent PCI (75.0%; *n* = 120/188) and coronary artery bypass graft (81.8%; *n* = 9/11; *P* > .999).

### 3.1 Survival analyses

**Figure 2:**
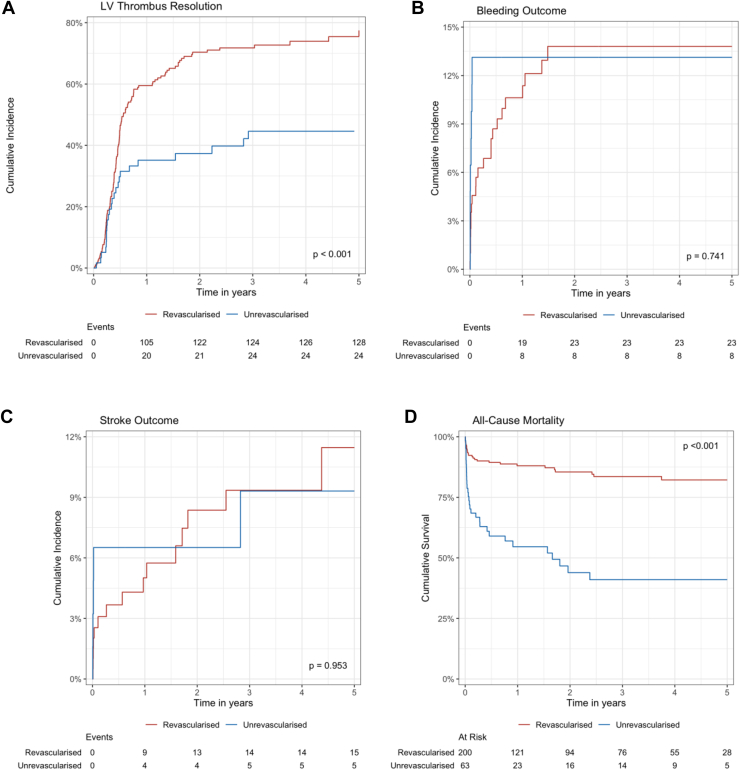
(A–D) Cumulative incidence function of outcomes and Kaplan–Meier estimates of mortality of postacute myocardial infarction left ventricular (LV) thrombus patients stratified by revascularization status.

Survival analyses (Figure 2A–D) were consistent with univariate analysis, which found that unrevascularized patients had lower incidence of LVT resolution in the cumulative incidence function (*P* < .001) and higher incidence of all-cause mortality in the Kaplan–Meier estimate (*P* < .001). There were no significant differences in bleeding (*P* = .795) and stroke/TIA outcomes (*P* > .999).

In competing risk regression analysis, revascularization was associated with LVT resolution in univariate analysis (HR, 2.14; 95% CI, 1.36-3.36; *P* < .001) but not in multivariable analysis (adjusted HR [aHR], 1.25; 95% CI, 0.74-2.13; *P* = .40; Table 2). At least dual therapy was associated with LVT resolution in both univariate (HR, 2.57; 95% CI, 1.66-3.96; *P* < .001) and multivariate (aHR, 1.84; 95% CI, 1.14-2.96; *P* = .01) analyses.

**Table 2:** Competing risk regression analysis for left ventricular thrombus resolution in postacute myocardial infarction left ventricular thrombus patients.

Variables	Univariate analysis			Multivariable analysis	
	*N*	HR (95% CI)	*P* value	aHR (95% CI)	*P* value
Age (per y)	263	0.98 (0.96-0.99)	**.002**	0.99 (0.97-1.00)	.168
Female sex	263	0.74 (0.46-1.18)	.21	1.04 (0.65-1.66)	.87
Anemia	263	0.57 (0.38-0.88)	**.01**	0.87 (0.54-1.42)	.586
Anterior STEMI	263	1.62 (1.06-2.47)	**.025**	0.93 (0.57-1.52)	.787
LVEF (per % increase)	263	1.04 (1.02-1.05)	**<.001**	1.02 (1.01-1.04)	**.003**
Revascularized	263	2.14 (1.36-3.36)	**<.001**	1.25 (0.74-2.13)	.40
Antithrombotic therapy	263		**<.001**		**.01**
Suboptimal therapy		(Reference)		(Reference)	
At least ACC + P2Y12		2.57 (1.66-3.96)		1.84 (1.14-2.96)	

ACC, anticoagulation; aHR, adjusted hazard ratio; HR, hazard ratio; LVEF, left ventricular ejection fraction; STEMI, ST-segment elevation myocardial infarction.

In multivariable Cox proportional hazards regression analysis, absence of revascularization (aHR, 2.30; 95% CI, 1.09-4.85; *P* = .03), female sex (aHR, 2.89; 95% CI, 1.31-6.36; *P* = .01), and absence of LVT resolution (aHR, 6.06; 95% CI, 2.99-12.3; *P* < .001) were independently associated with higher all-cause mortality after adjusting for age, anemia, anterior STEMI, and LVEF (Table 3). Revascularization status was not associated with bleeding or stroke outcomes in post-AMI LVT patients (Supplementary Table).

**Table 3:** Cox proportional hazards regression analysis for all-cause mortality in postacute myocardial infarction left ventricular thrombus patients.

Variables	Univariate analysis		Multivariable analysis	
	HR (95% CI)	*P* value	aHR (95% CI)	*P* value
Age (per y)	1.05 (1.03-1.07)	**<.001**	1.01 (0.99-1.04)	.34
Female sex	2.56 (1.40-4.67)	**.005**	2.89 (1.31-6.36)	**.01**
Anemia	0.36 (0.22-0.61)	**<.001**	1.34 (0.63-2.82)	.446
Anterior STEMI	3.42 (2.05-5.71)	**<.001**	1.22 (0.64-2.35)	.547
LVEF (per % decrease)	0.93 (0.90-0.95)	**<.001**	0.93 (0.90-0.97)	**<.001**
Unrevascularized	4.54 (2.72-7.59)	**<.001**	2.30 (1.09-4.85)	**.03**
No LVT resolution	9.61 (4.97-18.6)	**<.001**	6.06 (2.99-12.3)	**<.001**

aHR, adjusted hazard ratio; HR, hazard ratio; LVEF, left ventricular ejection fraction; LVT, left ventricular thrombus; STEMI, ST-segment elevation myocardial infarction.

## 4 Discussion

The major findings of this study are as follows: (1) at least dual therapy, but not revascularization status, was independently associated with LVT resolution, and (2) absence of LVT resolution and unrevascularized status were independently associated with all-cause mortality after adjusting for age, anemia, anterior STEMI, and LVEF.

### 4.1 Antithrombotic therapy and LVT resolution

The role of anticoagulation therapy in LVT treatment has been established by multiple existing studies, which demonstrated a decrease in embolic events and improvements in LVT resolution with anticoagulation [24,25]. Current cardiology society guidelines recommend an initial period of anticoagulation of 3 to 6 months in post-AMI patients with concomitant LVT [14,26]. This is then followed by interval imaging to determine LVT resolution, which would guide the need for prolonged therapy. In this cohort, being on at least dual therapy with anticoagulation and a P2Y12 inhibitor was associated with LVT resolution, in keeping with this recommendation. What is in contention, however, is the duration of triple therapy in this population of patients. A statement from American Heart Association recommends between 1 and 4 weeks of triple therapy before transitioning to dual therapy [13], while a state-of-the-art review from Journal of the American College of Cardiology suggests that the duration of triple therapy should be determined on a case-by-case basis evaluating the patient’s bleeding and ischemic risk, as well as ejection fraction (EF) recovery [11]. The former acknowledges that the recommendation on duration of triple therapy is based on studies of patients who had undergone PCI with coexisting atrial fibrillation, with none of said studies addressing patients with LVT. Further study on optimal antithrombotic therapy in this group of patients is warranted.

### 4.2 Revascularization and LVT resolution

In the pPCI era, undergoing revascularization may lead to improvement in LV dysfunction, thus disrupting the milieu that promotes LVT formation in the post-AMI setting. This was thought to increase the likelihood of LVT resolution and, hence, reduce the risk of stroke [27]. A prospective observational study by Rehan et al. [28] studied the incidence of post-AMI LVT in a population of STEMI patients who underwent pPCI and were treated with glycoprotein IIb/IIIa inhibitors and observed that there was a decrease in incidence compared with the prereperfusion era; however, no comparison was made between those who did or did not undergo revascularization. Similarly, most studies in the contemporary era examining first anterior STEMI patients who underwent PCI and were thereafter diagnosed with post-AMI LVT also reported an incidence of LVT much lower than those previously reported in the prereperfusion era [29,30]. Patients who did not undergo revascularization were likewise not included in these studies. A 1993 meta-analysis of patients with post-MI LVT found a trend toward a decrease in embolization in patients treated with thrombolytic therapy, suggesting that revascularization via thrombolysis in AMI may improve embolic outcomes in post-AMI, but this failed to achieve significance [31].

In this cohort, revascularization status did not appear to be independently associated with LVT resolution or stroke. While pPCI is indicated in the treatment of AMI, it may not have led to the improvement of LV dysfunction in all cases of MI [[32], [33], [34]]. First, the benefit of revascularization in myocardial salvage varies with the proportion of viable and nonviable myocardium at the time of pPCI [35,36], which may be attenuated in this cohort of post-AMI patients, as suggested by the presence of subsequent LVT formation. Second, a significant minority of post-AMI patients with LVT sustained non–ST-elevation MI, for whom timing of revascularization would be more variable, with resultant discrepancy in myocardial recovery. Hence, despite pPCI being a standard of care treatment for AMI, revascularization was not found to be independently associated with LVT resolution in this cohort.

### 4.3 Mortality in the post-MI LVT population

Despite its lack of association with LVT resolution, revascularization status remained predictive of all-cause mortality, in keeping with its role in the management of AMI [14,37,38]. The presence of LVT already signifies a sicker patient, which is more common in anterior MI and patients with poorer heart function. Patients did not undergo revascularization procedures due to medical comorbidities that rendered them unfit for revascularization or in view of patient preferences. While specifics of the rationale of patients declining these procedures were not available in this data set, a fraction of this population may likely have been fit for revascularization procedures, albeit with more comorbidities and, consequently, a higher risk quoted during informed consent, dissuading them from the procedure. Not all elements of this increased comorbidity burden would likely have been included in the multivariate model, such as chronic kidney disease. This likely contributes to the difference seen in terms of all-cause mortality in patients who were revascularized compared with those who were unrevascularized, despite the lack of association with LVT resolution.

Absence of LVT resolution was also found to be independently associated with all-cause mortality. In patients with LVT, LVT resolution has been shown to be a good prognostic factor. Kim et al. [27] examined patients with EF <50% who were diagnosed with LVT and found LVT resolution to be independently associated with a lower risk of the primary outcome of death, stroke, TIA, and arterial thromboembolic events (HR, 0.45; 95% CI, 0.21-0.98; *P* = .045). The study also found that EF improvement failure was significantly associated with absence of LVT resolution and LVT recurrence. In the post-MI LVT demographic, a prospective study examining patients who developed postacute coronary syndrome LVT, LVT resolution was found to be associated with a 6-fold decrease in all-cause mortality (HR, 5.59; 95% CI, 1.07-29.07; *P* = .04) [8]. This study adds to the body of evidence of the importance of LVT resolution in terms of patient-centered outcomes. This further underscores the importance of triple therapy in patients with post-MI LVT.

### 4.4 Strengths and limitations

To the best of our knowledge, this is the first study in the contemporary pPCI era that directly compared the outcomes of post-AMI LVT patients stratified by revascularization. Second, the study was conducted in a multiethnic cohort, with a large majority (93.5%; *n* = 246) being of Asian ethnicity, which addressed a knowledge gap in the existing literature of predominantly Caucasian cohorts. Lastly, the cohort size of post-AMI LVT patients was 263, which is a sizable number and improves the generalizability of the results.

We acknowledge the following limitations of the study. First, as a single-center retrospective cohort study, we were only able to draw correlations but not causative links. Second, the study involved a review of echocardiographic data, which, while specific, is not as sensitive as cardiac MR imaging for LVT [9] and may have missed out on smaller thrombi. The echocardiogram was also done primarily for routine post-AMI assessment and not specifically for LVT; hence, contrasted echocardiography was not performed up front. Nevertheless, echocardiography is easily accessible and is routinely performed in post-AMI patients to evaluate potential post-MI complications per guidelines [13,14] and would reflect the real-world incidence of post-AMI LVT formation closely. Third, interval imaging data such as LVEF were not consistently available and were not included in the multivariable regression models, which would have been useful in informing about the effects of revascularization and/or optimal medical therapy on post-AMI LV function recovery and clinical outcomes. Lastly, inclusion of LVT resolution status, a potential mediator, in the multivariable analysis for all-cause mortality may introduce over adjustment bias.

## 5 Conclusion

Revascularization status was not independently associated with the outcomes of LVT resolution, bleeding, or stroke but was associated with improved all-cause mortality in post-AMI patients with concomitant LVT, while the use of at least dual therapy was associated with increased LVT resolution and, in turn, lower all-cause mortality. Hence, optimal medical therapy, including early initiation and ensuring adherence, remains the mainstay of treatment in post-AMI patients who develop LVT.
