# Supplementary material for: Impact of coronary revascularization on clinical outcomes of postacute myocardial infarction patients with left ventricular thrombus
Source: Res Pract Thromb Haemost. 2025 May 21;9(4):102897. doi: 10.1016/j.rpth.2025.102897 (PMC12210294; doi:10.1016/j.rpth.2025.102897)
Supplement: Supplementary Table [file mmc1.docx]

Supplementary Table 1. Multivariable time-to-event analysis for bleeding and stroke outcomes in post-AMI LVT patients

| **Variables** | **Bleeding Outcome** | | **Stroke Outcome** | |
| --- | --- | --- | --- | --- |
|  | **aHR(95% CI)**^1^ | ***p*-value** | **aHR(95% CI)**^1^ | **p-value** |
| Age (per year) | 1.01 (0.98 to 1.04) | 0.410 | 0.99 (0.94 to 1.04) | 0.700 |
| Female sex | 0.49 (0.15 to 1.61) | 0.240 | 0.42 (0.04 to 3.93) | 0.440 |
| Anemia | 2.03 (0.91 to 4.52) | 0.084 | 0.79 (0.23 to 2.65) | 0.700 |
| Anterior STEMI | 0.65 (0.28 to 1.53) | 0.330 | 1.10 (0.22 to 5.43) | 0.910 |
| Unrevascularized | 0.95 (0.36 to 2.46) | 0.910 | 1.07 (0.29 to 3.98) | 0.930 |
| LVEF (per % increase) | 1.01 (0.98 to 1.04) | 0.550 | 0.99 (0.95 to 1.03) | 0.570 |
| Anti-thrombotic therapy |  | 0.610 |  | 0.470 |
| At least ACC + P2Y12 | (Reference) |  | (Reference) |  |
| Suboptimal therapy | 0.81 (0.35 to 1.85) |  | 1.44 (0.54 to 3.84) |  |

Abbreviations: ACC - anticoagulation; CI – confidence interval; aHR – adjusted hazard ratio; LVEF – left ventricular ejection fraction; STEMI – ST-segment elevation myocardial infarction
